# Paraphilias—where do we go from here?

**DOI:** 10.1038/s41443-024-00885-8

**Published:** 2024-06-05

**Authors:** Safiye Tozdan

**Affiliations:** Institute for Sex Research, Sexual Medicine and Forensic Psychiatry, University Medical Center Hamburg-Eppendorf, Martinistraße 52, D-20246 Hamburg, Germany

**Keywords:** Diagnosis, Therapeutics

The following is a quote of a man who had an exclusive sexual interest in children as long as he could think. He started a therapy and noticed over time that his sexual interest was more flexible than he had thought in the first place.

*“If I stick to my own experience, then one can certainly grow beyond a pedophilic orientation […] and become open to certain forms of adult sexuality. […] Pedophilia is by no means as firmly anchored […] as [some experts] claim. With this in mind, I would also like to encourage other affected people not to give up hope too quickly […].”*

The quote is an excerpt from a post by the man on the German platform www.schicksal-und-herausforderung.de [translated: fate and challenge] which addresses individuals with sexual interest in children. He was one of the first administrators of the website which clearly speaks against sexual contact with children. I found this post at the very beginning of my research activity and in addition to many other factors, it influenced my view of pedophilia and, in the broadest sense, also my view of other paraphilias that I have today. Through a variety of research efforts, the field has become very differentiated in recent years regarding the question whether a sexual interest in children is changeable or not. There are indications that this question may be an individual matter as pedophilic men describe their sexual interest in children as more or less flexible [1]. Additionally, there are on the one hand people who report changes of their sexual interest in children, and on the other hand, there are those who describe a rather fixed pedophilic interest over their lifespan [2]. I chose to include the quote above in this editorial as it reflects how the concepts of paraphilias not only change among researchers and society, but also among those who are affected by them—all three interacting with each other in the best case.

Paraphilias and paraphilic behavior appear to be rooted in human history and the past has shown that the concepts of paraphilias, their theoretical basis, and their treatment options are constantly changing. For example, at the very beginning, every sexual orientation that was not heterosexual was regarded as an illness [3]. Over time, science and society had to learn to distinguish between paraphilic interest, paraphilic behavior, and paraphilic disorder as well as between non-harmful sexual interests and sexual behaviors that are a threat to others and/or imply criminal acts. Thus, diagnostic criteria have always been discussed and criticized by experts and are then further developed. Similarly, stereotypes must always and constantly be overcome, like for example the idea that only men can have a pedophilic interest. Because of this assumption, pedophilia in females has been neglected in research for a long time giving the impression that it rarely exists in women. Indeed, recent research addressing this topic has been shown that women with sexual interests exist and that they have a problem in being seen as they perceive themselves as minority within a minority [4]. Taking into account these constant changes over time, the question that might arise is where does it take us in the future?

This editorial introduces a special issue on paraphilias. The first contribution summarizes the historical development of paraphilias as well as treatment options and presents the current diagnostic criteria for paraphilias in the “International Statistical Classification of Diseases and Related Health Problems” (ICD) as well as the “Diagnostic and Statistical Manual of Mental Disorders” (DSM) [5]. The next two studies shed light on special topics. Turner et al. investigated the prevalence of paraphilias in adults with attention deficit/hyperactivity disorder and examined the association between paraphilias and hypersexuality in this sample as well [6]. Kurvits et al. conducted a study capturing data on hypersexual behavior and paraphilic interest among adult patients with chronic tic disorders and Tourette syndrome [7]. The following article by Haung et al. deals with aspects of BDSM and investigates associations between sexually submissive and dominant behaviors and sexual dysfunction analyzing three population-based samples [8]. The next two studies provide insides in how men and women with sexual interest in children think and behave. Yoon et al. present psychometric properties of explicit and implicit measures for emotional congruence with children among a sample of adult community males [9]. Tozdan et al. examined qualitative data of women who have a sexual interest in children revealing how they think about the cause of their sexual interest in children as well as experiences with disclosure and professional help [10]. Afterward, two articles address the topic of child sexual abuse. Analyzing qualitative interviews by men convicted of child sexual abuse, Knack et al. explored the narratives these men constructed around their subjective motivations for offending [11]. Jordan et al. investigated data on subjective sexual arousal after being presented with experimental sexual stimuli among a diverse male sample [12]. The last four contributions are dedicated to the treatment of paraphilias. First, Galizia et al. provide a broad overview of treatment approaches related to problematic sexual fantasies [13]. Second, Shumate et al. collected insights on treatment modalities for paraphilic disorders, psychopathy, and those who sexually offend [14]. Third, Casademont et al. describe “The Hamburg Youth Prevention Project” which addresses a special population with sexual interest in children, namely adolescents [15]. And fourth, De Tribolet-Hardy et al. have contributed a perspective on the clinical care of pedophilic individuals in Switzerland [16].

These contributions reflect the diversity of perspectives on the topic of paraphilias leading to important recent developments in the research field. Therefore, I would like to sincerely thank all the authors of this issue for their valuable contributions and the committed cooperation. My special thanks also go to the Editor-in-Chief Ege Can Serefoglu et al. for the enriching collaboration. It was a pleasure and an honor to be a guest editor for the International Journal of Impotence Research. I hope that this special issue inspires researchers in the field of sexual medicine to think ahead and to realize new research ideas continuing the collaborative work of understanding individuals with paraphilias and paraphilic behaviors as well as their relations to other aspects of human characteristics and behaviors.
